# Oral pathobiont induces systemic inflammation and metabolic changes associated with alteration of gut microbiota

**DOI:** 10.1038/srep04828

**Published:** 2014-05-06

**Authors:** Kei Arimatsu, Hitomi Yamada, Haruna Miyazawa, Takayoshi Minagawa, Mayuka Nakajima, Mark I. Ryder, Kazuyoshi Gotoh, Daisuke Motooka, Shota Nakamura, Tetsuya Iida, Kazuhisa Yamazaki

**Affiliations:** 1 Laboratory of Periodontology and Immunology, Division of Oral Science for Health Promotion, Niigata University Graduate School of Medical and Dental Sciences, Niigata, Japan; 2 Division of Periodontology, Department of Oral Biological Science, Niigata University Graduate School of Medical and Dental Sciences, Niigata, Japan; 3 Division of Periodontology, Department of Orofacial Sciences, School of Dentistry, University of California, San Francisco, USA; 4 Department of Infection Metagenomics, Research Institute for Microbial Diseases, Osaka University, Osaka, Japan

## Abstract

Periodontitis has been implicated as a risk factor for metabolic disorders such as type 2 diabetes, atherosclerotic vascular diseases, and non-alcoholic fatty liver disease. Although bacteremias from dental plaque and/or elevated circulating inflammatory cytokines emanating from the inflamed gingiva are suspected mechanisms linking periodontitis and these diseases, direct evidence is lacking. We hypothesize that disturbances of the gut microbiota by swallowed bacteria induce a metabolic endotoxemia leading metabolic disorders. To investigate this hypothesis, changes in the gut microbiota, insulin and glucose intolerance, and levels of tissue inflammation were analysed in mice after oral administration of *Porphyromonas gingivalis*, a representative periodontopathogens. Pyrosequencing revealed that the population belonging to Bacteroidales was significantly elevated in *P. gingivalis*-administered mice which coincided with increases in insulin resistance and systemic inflammation. In *P. gingivalis*-administered mice blood endotoxin levels tended to be higher, whereas gene expression of tight junction proteins in the ileum was significantly decreased. These results provide a new paradigm for the interrelationship between periodontitis and systemic diseases.

Periodontal diseases are mainly chronic infectious diseases result from response to a complex dental plaque microbiome containing various periodontopathic bacteria species. Periodontal diseases destroys the tooth-supporting tissues and leads to tooth loss if not adequately treated. Advanced form of the diseases affects ~10% to 15% of adults worldwide^1^.

Epidemiological evidence suggests that periodontal infection is associated with an increased risk of a variety of diseases such as atherosclerotic vascular diseases^2^,^3^, type 2 diabetes^4^,^5^, and non-alcoholic fatty liver disease^6^. Among the various periodontopathic bacteria, considerable research has focused on the role of *Porphyromonas gingivalis* in possible mechanisms linking periodontal diseases and other human diseases, due to its unique pathogenicity^7^ and its association with these various diseases.

Once an inflammatory lesion in the periodontium is established, bacteria from the dental plaque can invade into the gingival tissue through the ulcerated sulcular epithelial lining of periodontal pockets and then disseminate into the systemic circulation^8^. In addition, various proinflammatory cytokines are produced in the inflamed periodontal tissue^9^ which can also enter the systemic circulation. Although the precise mechanisms have not been clarified, the resulting bacteremia and increase in circulating inflammatory mediators may potentiate an inflammatory reaction in other tissues or organs. In fact, a number of studies have demonstrated elevated levels of serum hs-CRP^10^ and IL-6^11^,^12^ in periodontitis patients when compared with periodontally healthy subjects. By contrast, there is little evidence for an increased detection and/or numbers of oral bacteria in the blood of periodontitis patients when compared with periodontally healthy subjects^13^. In addition, we have shown that repeated oral inoculation of *P. gingivalis* induced elevation of serum inflammatory markers (serum amyloid A and IL-6) in mice. However, no *P. gingivalis* was detected in the blood at any time point of these experiments^14^. Moreover, in this mouse model, a limited inflammatory cell infiltrate was seen in the gingival tissue.

It is well-known that obesity, a chronic disease characterized by an excessive growth of adipose tissue, increases the risk of insulin resistance, diabetes, atherosclerosis, hypertension, chronic kidney disease, and cardiovascular morbidity and mortality^15^,^16^,^17^. Interestingly, many of these diseases are also associated with inflammatory periodontal diseases. Insulin resistance, a key feature of obesity, is a state in which the sensitivity of target cells to respond to ordinary levels of insulin is reduced. This insulin sensitivity is modulated in part by adipokines. In obesity, adipokine production is either up-regulated or down-regulated. Specifically, insulin resistance-associated adipokines are usually up-regulated whereas insulin-sensitivity-associated adipokines are down-regulated^18^.

Obesity is reported to be associated with periodontal diseases. Saito *et al*. first reported that obese Japanese subjects were more likely to have periodontal disease than thin subjects^19^. Although later studies supported the positive association between obesity and periodontitis^20^,^21^,^22^, there are also studies showing no association between these two conditions^23^. While there are several epidemiological studies demonstrating this association, there are few reports on the mechanism linking obesity and periodontal diseases. One proposed mechanism involves the adipocyte hypertrophy found in obesity. This adipocyte hypertrophy leads to infiltration of macrophages and other inflammatory cells. This phenomenon results in low-grade chronic inflammation and enhanced proinflammatory cytokine production^24^,^25^. Among the many adipokines, TNF-α is considered a key player in inflammatory cell activation and recruitment^26^. TNF-α produced by the adipocytes together with TNF-α produced in the periodontal tissue may induce further periodontal tissue destruction. However, several reports have shown that serum TNF-α levels of periodontitis patients are either not elevated or lower in periodontitis patients compared with non-periodontitis subjects^27^. Thus, the pathogenic mechanisms for the effects of periodontal disease affecting systemic disease and the specific effects of the infection with periodontopathic bacteria on the pathology of various tissues remain to be elucidated.

In this study, we propose that endotoxemia is responsible for inflammation of various organs and tissues in this mouse model through *P. gingivalis* oral administration. However, this endotoxemia may not be induced directly by bacteria from the oral cavity, but rather through changes in gut microbiota induced by inoculation of bacteria from the oral cavity into the gut through swallowing. Subsequent activation of proinflammatory genes within blood vessels, adipose tissue and liver could increase the risk of atherosclerosis, insulin resistance and non-alcoholic fatty liver disease (NAFLD), respectively.

## Results

### Local and systemic inflammation in *P. gingivalis*-administered mice

**Figure 1:**
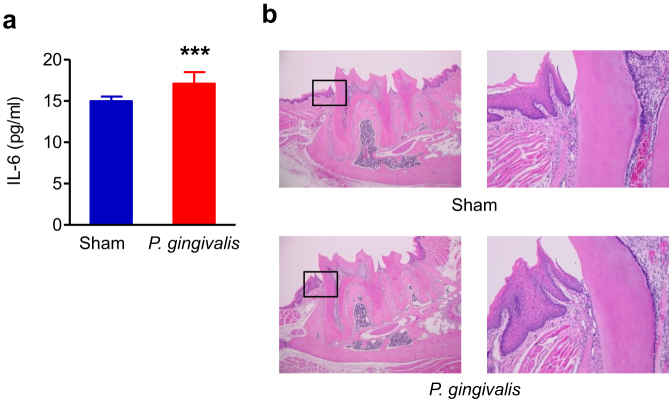
Systemic and local inflammation in *P. gingivalis*-administered and sham-administered mice (N = 8 in each group). (a) Serum level of IL-6. All data are means ± SD. Significant differences were observed between the *P. gingivalis*-administered groups and the sham-administered group (***p < 0.001, Mann-Whitney U-test). (b) Histological findings of gingival tissues of *P. gingivalis*-administered and sham-administered mice. Sections of the periodontium around the disto-buccal root of the first molar were H-E stained. Right panels are magnified views of the boxed areas. No difference of gingival inflammation was observed between *P. gingivalis*-administered and sham-administered mice.

Our previous study and others demonstrated that repeated oral gavage administration of *P. gingivalis* induced alveolar bone resorption. However, whether this could be due to local inflammation of gingival tissue is still unresolved. Although serum IL-6 levels significantly increased in *P. gingivalis*-administered mice compared with sham-administered mice, no difference of gingival inflammation, which was minimal, between *P. gingivalis*-administered and sham-administered mice was observed (Fig. 1).

### *P. gingivalis* administration induces insulin resistance, liver steatosis, and macrophage infiltration in adipose tissue

**Figure 2:**
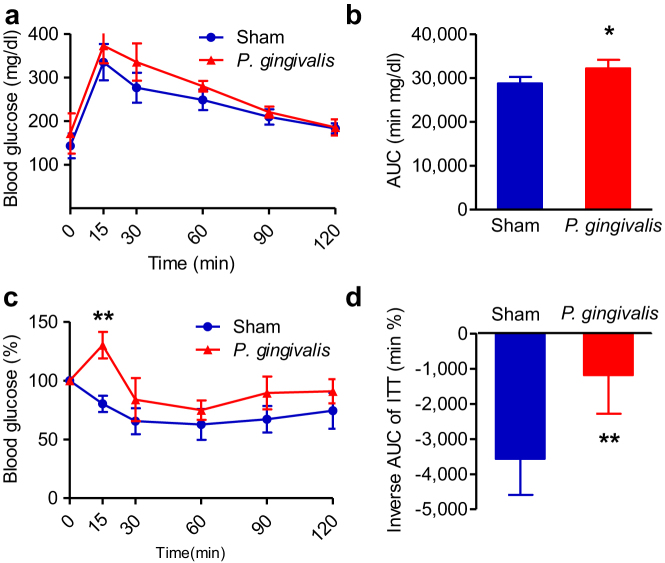
*P. gingivalis* induced insulin resistance. (a) Intraperitoneal glucose tolerance test. Blood glucose levels were determined at the indicated times after intraperitoneal load of glucose (1 g/kg). (b) Area under blood concentration curve (AUC) of the Fig. 2a. (c) Intraperitoneal insulin tolerance test (ITT). Blood glucose levels were determined at the indicated times after intraperitoneal load of insulin (0.5 unit/kg). (N = 6 in each group). (d) Insulin sensitivity was assessed by inverse AUC of ITT. All data are means ± SD. (*p < 0.05; **p < 0.01, Mann-Whitney U-test).

**Figure 3:**
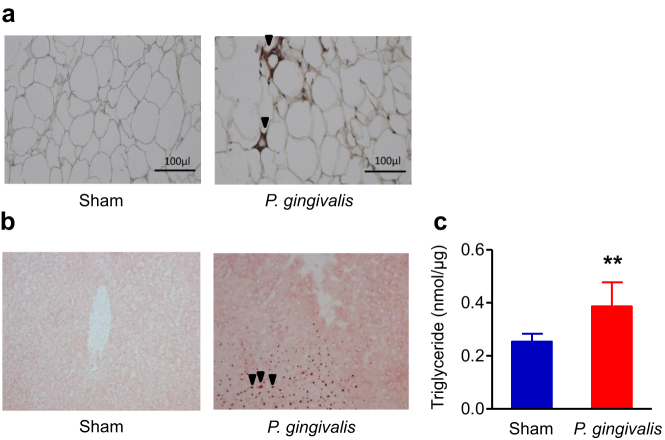
Relationship between *P. gingivalis* administration and inflammatory changes of epididymal adipose tissue and liver. (a) Epididymal adipose tissues were fixed 10% formalin, sectioned, and stained with a rat anti-mouse F4/80 primary antibody (Abd Serotec). Arrow heads indicate F4/80 positive macrophages. (b) Oil red O staining of the liver tissue are shown. Increased number of lipid containing hepatocytes (arrow heads) are seen in *P. gingivalis*-administered mice. (c) *P. gingivalis* administration increased hepatic triglyceride content. (N = 8 in each group). All data are means ± SD. (**p < 0.01, Mann-Whitney U-test).

To explore the effects of oral gavage administration of *P. gingivalis* on insulin resistance, we performed glucose and insulin tolerance tests. As shown in Fig. 2, both glucose tolerance and insulin sensitivity was diminished in *P. gingivalis*-administered mice compared with sham-administered mice, despite similar body weight changes during the experimental period (Supplementary Fig. S1). Although the effect of *P. gingivalis* administration was relatively weak, a significant difference was observed at 15 min for the insulin tolerance test, and an overall effect was evident as shown in Figs. 2b and 2d. However, there were no differences in blood insulin levels between *P. gingivalis*-administered mice and sham-administered mice (Supplementary Fig. S2). Oral gavage administration of *P. gingivalis* promoted macrophage infiltration into the adipose tissue and a typical crown-like structure was observed histologically (Fig. 3a). Histological analysis showed that *P. gingivalis*-administered mice had accumulated much higher amounts of hepatic fat (Fig. 3b) and triglyceride (Fig. 3c), as compared to the sham-administered mice.

### *P. gingivalis* administration induces an inflammatory response in adipose tissue

**Figure 4:**
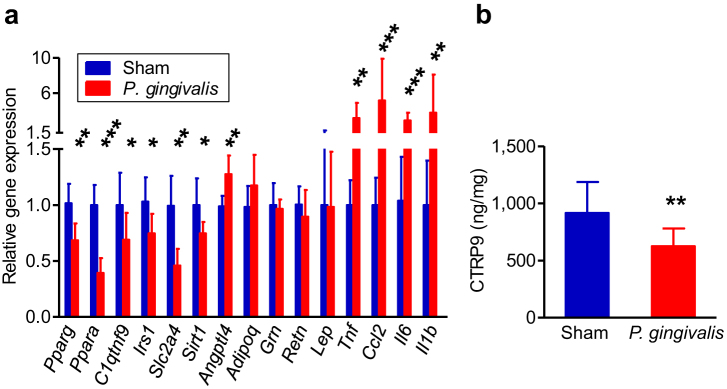
Effect of oral administration of *P. gingivalis* on the gene and protein expressions in epididymal adipose tissue. (a) Comparison of relative gene expression levels in the adipose tissue between the *P. gingivalis*-administered and the sham-administered mice (N = 8 in each group). The relative quantity of experimental mRNA was normalized to the relative quantity of glyceraldehyde-3-phosphate dehydrogenase (GAPDH) mRNA. (b) CTRP9 contents in the epididymal adipose tissue were determined by ELISA of tissue lysates. All data are means ± SD. (*p < 0.05; **p < 0.01; ***p < 0.001, Mann-Whitney U-test).

Next we compared gene expression profiles of epididymal adipose tissue between *P. gingivalis*-administered and sham-administered mice (Fig. 4). In *P. gingivalis*-administered mice, expression of proinflammatory genes *Tnfα*, *Ccl2*, *Il6*, and *Il1β* were significantly upregulated, whereas the genes that improve insulin sensitivity *Pparγ, Pparα, C1qtnf9, Irs1, Sirt1*, and *Slc2a4* were downregulated. In addition to proinflammatory genes, expression of *Angptl4* which is involved in insulin resistance was also upregulated. These results suggest that oral gavage administration of *P. gingivalis* induces an inflammatory response and insulin resistance in adipose tissue. There was no difference in the expression of three regulators of insulin signalling, *Grn*, *Retn*, and *Lep*, between *P. gingivalis*-administered mice and sham-administered mice.

### *P. gingivalis* administration induces an inflammatory response in the liver and an inflammation-related gene response

**Figure 5:**
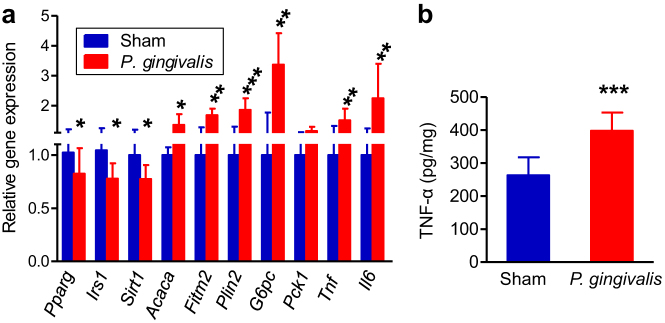
Effect of oral administration of *P. gingivalis* on the gene and protein expressions in liver. (a) Comparison of relative gene expression levels in the liver tissue between the *P. gingivalis*-administered and the sham-administered mice (N = 8 in each group). The relative quantity of experimental mRNA was normalized to the relative quantity of glyceraldehyde-3-phosphate dehydrogenase (GAPDH) mRNA. (b) TNF-α levels in the liver tissue were determined by ELISA of tissue lysates. All data are means ± SD. (*p < 0.05; **p < 0.01; ***p < 0.001, Mann-Whitney U-test).

Oral administration of *P. gingivalis* led to increased mRNA expression of the proinflammatory cytokines TNF-α and IL-6, as well as of *Fitm2* and *Plin2*, both of which are strongly associated with lipid droplet formation in the liver. Furthermore, *Acaca* and *G6pc*, which positively regulate fatty acid synthesis and gluconeogenesis respectively, were also upregulated. On the other hand, mRNA expression of the molecules having potentially anti-inflammatory properties *Pparγ* and *Sirt1*, were downregulated. In addition, expression of the insulin signalling gene *Irs1* was also decreased compared with sham-administered mice. We observed that not only gene expression (Fig. 5a) but also protein levels of TNF-α were increased in the liver of *P. gingivalis*-administered mice (Fig. 5b).

### Oral administration of *P. gingivalis* alters the gut microbial ecology

**Figure 6:**
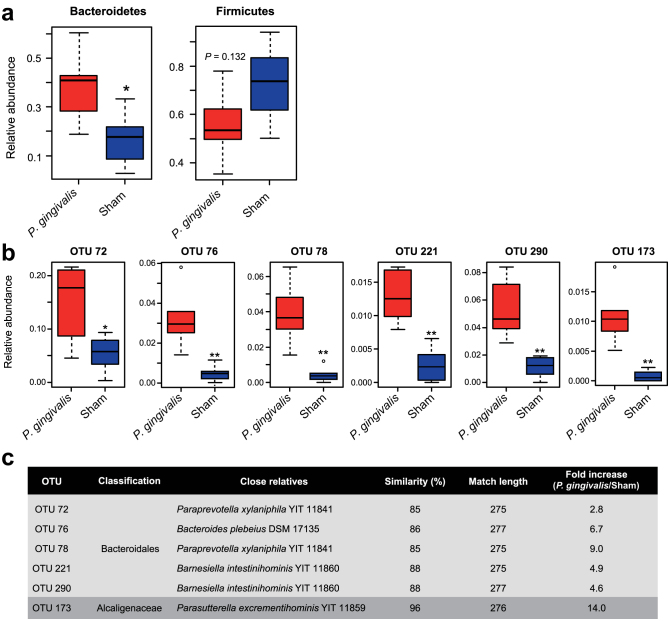
Comparison of the gut microbiota between *P. gingivalis*-administerd and sham-administered mice by 16S rRNA sequencing analysis. Relative abundances of each bacterial group in Phylum (a) and OTU (b) level are indicated by boxplot. Each defined OTU obtained from 16S rRNA deep sequencing was compared to the genome database (GenomeDB) from NCBI using blastin (c). Close relative species and percent similarities are shown. Fold increases indicate the mean ratio of relative abundance of the OTU in the gut microbiota from the *P. gingivalis*-administered group to sham-administered group. (*p < 0.05, **p < 0.01, Mann-Whitney U-test).

After 10 cycles of oral administration of live *P. gingivalis* W83, the contents of the distal 3cm of the small intestine (ileum) were recovered immediately after euthanization by manual extrusion. We defined the extent to which oral administration of *P. gingivalis* altered the composition of the microbiota by pyrosequencing the 16S ribosomal RNA genes in the ileum contents. In total, 41,557 (mean 6,926 reads/sample) and 32,108 (mean 5,351 reads/sample) sequencing reads for the *P. gingivalis*-administered samples and the sham-administered samples, respectively, were obtained by two sequencing runs. The majority of aligned reads were determined to be from the phyla Firmicutes and Bacteroidetes, representing 55.4% and 38.7% of the flora respectively in *P. gingivalis*-administered mice, and 72.8% and 17.0%, respectively in sham-administered mice. The difference of the proportion of Bacteroidetes and Firmicutes between *P. gingivalis*-administered mice and sham-administered mice was statistically significant (Fig. 6a).

Notably, operational taxonomy unit (OTU)-based bacterial diversity analysis revealed that the population belonging to Bacteroidales was significantly elevated in *P. gingivalis*-administered mice compared with sham-administered mice (Fig. 6b and 6c). Because *P. gingivalis* belongs to the order Bacteroidales, increased proportions of the order Bacteroidales could be due to the administered *P. gingivalis*. To clarify this possible mechanism, DNA samples of the ileum contents were amplified using *P. gingivalis*-specific primers. However, no *P. gingivalis*-specific DNA was detected (Supplementary Fig. S3).

### *P. gingivalis* administration increases serum endotoxin level

**Figure 7:**
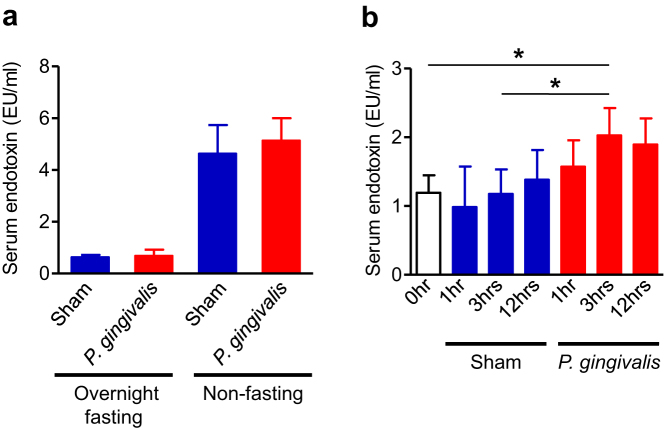
Effect of oral administration of *P. gingivalis* on the serum endotoxin levels. (a) Serum endotoxin (LPS) concentration (EU/ml) were determined after 10 cycles of *P. gingivalis* administration or sham administration (N = 8 in each group). All data are means ± SD. (b) Serum endotoxin (LPS) concentration (EU/ml) were determined at the indicated times after single administration of *P. gingivalis* (N = 6 in each group). All data are means ± SD. (*p < 0.05, one-way ANOVA).

As it has been reported that changes in the gut microbiota influence metabolic endotoxemia, we compared the serum endotoxin levels between *P. gingivalis*-administered mice and sham-administered mice. As shown in Fig. 7a, endotoxin levels were very low after overnight fasting, with no difference between *P. gingivalis*-administered mice and sham-administered mice. However, the endotoxin levels dramatically increased if the mice were fed ad libitum irrespective of *P. gingivalis* administration. The endotoxin level tended to be higher in *P. gingivalis*-administered mice compared to sham-administered mice. However, this difference was not statistically significant (Fig. 7a). In order to further examine the effect of oral administration of *P. gingivalis* on endotoxemia, the serum endotoxin levels were monitored at 1, 3, 12 hrs. after a single administration of *P. gingivalis*. Serum endotoxin levels increased as early as 1 hr. after administration, peaked at 3 hrs., and maintained these higher levels until 12 hrs. (Fig. 7b). One hour after oral administration, *P. gingivalis* was detected in the jejunum and ileum, and its proportion in the microbial flora decreased at 3 hrs. Although *P. gingivalis* DNA was detected in the colon at 16 hrs, the proportion of *P. gingivalis* DNA was extremely low because of far larger quantities of other bacteria in the colon (Supplementary Fig. S4). At these time points, no *P. gingivalis* was detected in the blood samples of *P. gingivalis*-adminstered mice whereas other bacterial DNA was detected (Supplementary Fig. S5).

### Changes in gene expression profiles in the intestine by oral administration of *P. gingivalis*

**Figure 8:**
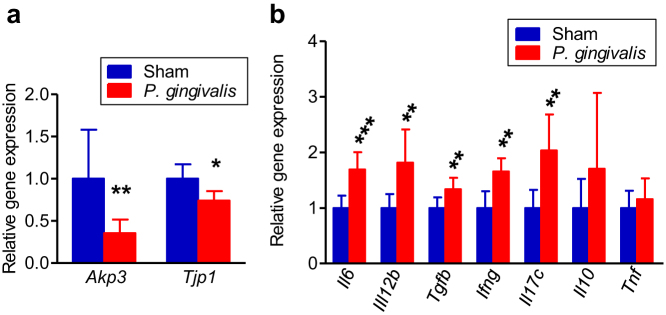
Comparison of relative gene expression levels in the small intestine (a) and colon tissue (b) between the sham-administered group and the *P. gingivalis*-administered group (N = 8 in each group). The relative quantity of experimental mRNA was normalized to the relative quantity of glyceraldehyde-3-phosphate dehydrogenase (GAPDH) mRNA. All data are means ± SD. (*p < 0.05; **p < 0.01, ***p < 0.001, Mann-Whitney U-test).

Next we examined the effect of *P. gingivalis* administration on the expression of genes that play important role for prevention of metabolic syndrome and metabolic endotoxemia in the small intestine (Fig. 8a) and inflammatory responses in the large intestine (Fig. 8b). The expression of genes that code intestinal alkaline phosphatase (*Akp3*) and tight junction protein (*Tjp1*) in the small intestine were both downregulated in *P. gingivalis*-administered mice compared to sham-administered mice. In the samples of large intestine, mRNA expression of the proinflammatory cytokines IL-6, IL-12β, IFN-γ and IL-17c were significantly upregulated in *P. gingivalis*-administered mice compared to sham-administered mice. However, there was no difference in expression of IL-10 and TNF-α.

## Discussion

Periodontal disease is a chronic inflammatory disease where groups of periodontopathic bacteria such as *P. gingivalis* plays a major role in its initiation and progression. A number of epidemiological studies have suggested that periodontal disease is a risk factor for various systemic diseases and conditions, including cardiovascular disease, type 2 diabetes, NAFLD, and rheumatoid arthritis^28^. Interestingly, obesity increases the risk of these diseases^29^,^30^,^31^. In addition, obesity is also associated with an increased risk of periodontal disease. Therefore, it is possible that periodontal disease and obesity have similar effects on these systemic conditions.

The common systemic effect of obesity and periodontal disease is considered to be low-grade inflammation^13^. It is well-known that diet-induced obesity is implicated in systemic low grade inflammation. Nutritional fatty acids activate Toll-like receptor 4 (TLR4) signalling in adipocytes and macrophages. In addition, the capacity of fatty acids to induce inflammatory signalling in adipocytes or macrophages is blunted in the absence of TLR4^32^,^33^. Furthermore, adipose tissue lipolysis from hypertrophied adipocytes, could serve as a naturally occurring ligand for TLR4 to induce inflammation. It is apparent that gut microbial ecology could be an important factor in the development of obesity by affecting energy homeostasis. For example, Cani *et al*. elegantly demonstrated that the composition of the gut microbiota is influenced by a high-fat diet- and genetically obese ob/ob-induced metabolic endotoxemia^34^. Components originating from the gut microbiota, such as lipopolysaccharide, lipoteichoic acid, peptidoglycan, flagellin, and bacterial DNA can cause immune system activation and subsequent inflammation.

Proposed mechanisms for periodontal diseases inducing systemic inflammation have included: (i) the direct effect of infectious agents or their products, and (ii) increased expression of cytokines, chemokines, and cell adhesion molecules produced in periodontitis lesions^35^. Additional mechanisms may include, (iii) translocation of swallowed *P. gingivalis* from the gut to the circulationg system, and (iv) alteration of gut microbial composition-induced increases in gut epithelial permeability by swallowed *P. gingivalis*. The first hypothesis is based on the lesion size of periodontal disease (periodontitis). It is reported that the mean dentogingival epithelial surface area of periodontitis patients where subgingival biofilm is contacting a thinning and/or ulcerated gingival sulcular epithelium is approximately 20 cm^2^ 36. This area is considered to act as an entrance of periodontopathic bacteria into the systemic circulation. This mechanism is supported by a number of studies that have demonstrated an association between periodontal disease and endotoxemia. However, periodontal treatment-induced endotoxemia is detectable as early as 5 min after instrumentation and disappears at 30 min^37^,^38^. In our study, increases in the serum endotoxin level was observed 1 hr after a single administration of *P. gingivalis* and peaked at 3 hrs. For the second hypothesis, there is no direct evidence that increased inflammatory markers in periodontitis patients are in fact derived from inflamed periodontal tissues. For the third possibility, although *P. gingivalis* was detected in the jejunal and ileal contents, and colonic contents up to 3 hrs and 16 hrs after a single administration, respectively, it was not detected in the blood of *P. ginigivalis*-administered mice whereas other bacterial DNA was detected in the blood. Therefore, it is highly likely that detected endotoxins are not derived from *P. gingivalis*.

Given that recent findings have implicated an altered gut microbiota as a contributor of not only metabolic diseases such as atherosclerosis^39^,^40^, type 2 diabetes^41^, and NAFLD^42^ but also immune diseases such as rheumatoid arthritis^43^ that are also associated with periodontitis, it is reasonable to assume that the systemic inflammatory changes seen in *P. gingivalis*-administered mice could be attributable to this altered gut microbiota. Furthermore, periodontal diseases themselves could be modulated by alteration of gut microbiota-induced systemic inflammation. In support of this hypothesis, it has been reported that high-fat diet-induced obesity increased alveolar bone resorption, a characteristic feature of periodontitis^44^,^45^.

The swallowed saliva of periodontitis patients is reported to contain up to 10^9^ bacteria/ml, in 1.0–1.5 L/day^46^,^47^,^48^, making a total of greater than 10^12^ bacteria/day. Since the bacterial flora of the oral cavity is distinct from that of the gut^49^, it is possible that swallowed bacteria could affect the composition of the gut microflora. In fact, it has been reported that oral probiotic intervention alters gut bacterial composition^50^. Thus, it is unlikely that oral bacteria alone could be causative agents of endotoxemia in periodontitis patients.

In the present study, oral administration of *P. gingivalis* induced a change of bacterial composition in the ileal microflora. Although several studies have shown the beneficial effect of Bacteroidetes phylum on the gut^51^,^52^, we observed an increased proportion of the order Bacteroidales belonging Bacteroidetes phylum, accompanied by insulin resistance and the alteration of gene expression in adipose tissue and liver. The expression of several proinflammatory genes and anti-inflammatory or insulin sensitivity-improving genes were upregulated or downregulated, respectively, in the adipose tissue and the liver of *P. gingivalis*-administered mice. In addition, expression of *Sirt1* which is reported to induce an increase in glucose uptake and insulin signalling was also downregulated. It has also been reported that SIRT1 expression is inversely related to inflammatory gene expression, particularly TNF-α. These changes in proinflammatory and anti-inflammatory gene expression may contribute to increases in serum glucose levels and in insulin intolerance. Although the effect of *P. gingivalis* is not robust, continuous deterioration of glucose metabolism could have significant effect.

One of the possible reasons for the discrepancy of inflammation-associated change of microbiota between previous studies and our study could be the difference of the site from where the samples were obtained. Previous studies analyzed faecal or caecal samples, whereas we analysed ileal contents. The ileum is considered to be an important organ because chylomicron is formed into small vesicles in the epithelial cells of the ileum, and lipid is mainly absorbed from the ileum. In addition, Peyer's Patches are located in the ileal wall. Therefore, as previously demonstrated with caecal bacterial^53^, the bacterial composition in the ileum may have significant impact on systemic inflammation. It is also noteworthy that in humans, Bacteroidetes may promote type 2 diabetes through an endotoxin-induced inflammatory response^54^.

Henao-Mejia *et al*., demonstrated that a significant expansion of Porphyrmonadaceae was found following HFD or with methionine-choline-deficient diet administration in the faecal microbiota in the inflammasome-deficient setting and was associated with progression of NAFLD and obesity^55^. We propose that administered *P. gingivalis* is not directly responsible for the increase of the “family” Porphyromonadaceae in the gut, as we did not detect *P. gingivalis* in the gut by using specific primers. However, bacteria belonging to this “family” of bacteria may play a role in the induction of endotoxemia and subsequent inflammatory responses. Alternatively, *P. gingivalis* may suppress inflammasome activation by other bacteria. For example, it has been reported that *P. gingivalis* suppresses inflammasome activity through inhibition of endocytosis^56^.

Another interesting finding from this study was that mRNA expression of alkaline phosphatase was downregulated in the ileum of *P. gingivalis*-administered mice. Kalannen *et al*. previously demonstrated that a defect in intestinal alkaline phosphatase (IAP), was associated with high-fat diet-induced metabolic syndrome, and endogenous and orally supplemented IAP inhibited endotoxin absorption, as well as reversed metabolic syndrome in mice^57^. Therefore, IAP is considered to play an important role in the suppression of endotoxemia. Although the underlying mechanisms by which oral administration of *P. gingivalis* and/or alteration of gut microbiota downregulate the mRNA of alkaline phosphatase has not been clarified, downregulation of the *Akp3* gene may be a factor for elevated systemic inflammation. Moreover, gene expression analysis of the small intestine demonstrated downregulated mRNA expression of the tight junction protein ZO-1 in *P. gingivalis*-administered mice. In previous studies on mouse models, the endotoxemia following high-fat diet administration was associated with reduced expression of genes encoding for ZO-1 and occludin^34^. These results suggest that administered *P. gingivalis* alters the gut microbiota, and alters the gut epithelial cell barrier function, resulting in increased gut permeability. However, the mechanism for how this change in the gut microbiota impairs gut barrier function has not been elucidated. *P. gingivalis* administration-induced alterations of the gut microbiota also induced upregulated mRNA expression of various proinflammatory cytokines. It is not known whether these inflammatory changes of the large intestine affect systemic inflammatory responses.

In conclusion, in the present study, we demonstrated that oral administration of *P. gingivalis* induced alteration of gut microbiota as well as inflammatory changes in various tissues and organs. These changes are considered to be attributable to increases in levels of endotoxin in the blood. However, as with previous observations on the influence of high-fat diet-induced metabolic endotoxemia induced changes of the gut microbiota, it remains to be elucidated whether a cause-and-effect relationship exists between oral administration of *P. gingivalis*-induced systemic inflammation and changes in the gut microbiota. Also, further investigations are needed to examine whether other oral bacteria have similar effects on the systemic metabolism. Because the composition of the oral microflora and gut microflora are quite distinct, the considerable flow of large quantities of oral bacteria into gut during the frequent act of swallowing could disturb the balance of the gut microflora. Furthermore, bacterial components responsible for the alteration of gut bacterial composition have also not been elucidated. Therefore, further studies are needed to clarify the exact mechanisms of how *P. gingivalis* induces shifts in the gut microbiota towards the production of metabolic products and shifts in the composition of bacterial species responsible for the induction of metabolic syndrome.

## Methods

### Mice

All experiments were performed in accordance with the Regulations and Guidelines on Scientific and Ethical Care and Use of Laboratory Animals of the Science Council of Japan, enforced on June 1, 2006, and approved by the Institutional Animal Care and Use Committee at Niigata University (permit number 39). Six-week-old male C57BL/6N mice were obtained from Japan SLC, Inc. (Shizuoka, Japan). The mice were acclimatized under specific pathogen-free conditions and fed regular chow and sterile water until the commencement of infection at 8 weeks of age.

### Bacterial cultures

*P. gingivalis* strain W83 was cultured in modified Gifu anaerobic medium (GAM) broth (Nissui, Tokyo, Japan) in an anaerobic jar (Becton Dickinson Microbiology System, Cockeysville, MD) in the presence of an AnaeroPackTM (Mitsubishi Gas Chemical Co. Inc., Tokyo, Japan) for 48 hours at 37°C. Bacterial suspensions were prepared in phosphate-buffered saline (PBS) without Mg^2+^/Ca^2+^ using established growth curves and spectrophotometric analysis. The number of CFUs was standardized by measuring optical density (600 nm).

### Oral administration

The murine experimental periodontitis model was developed according to Baker *et al*.^58^ with slight modifications. A total of 10^9^ CFU's of live *P. gingivalis* suspended in 100 μl of PBS with 2% carboxymethyl cellulose (Sigma-Aldrich, St. Louis, MO) was given to each mouse via a feeding needle. This suspension was given 2 times a week for 5 weeks. The control group was sham-administered without the *P. gingivalis*. During the experimental period, all mice were allowed to eat and drink ad libitum. One day after the final treatment, oral swabs were obtained and tested for the presence of *P. gingivalis* as previously described^59^. Mice were then euthanized with CO_2_, and their tissues were removed.

### Detection of *P. gingivalis* in blood and intestinal contents

Whole blood was taken at at 15 min, 3, 12, and 24 hrs after a single administration of *P. gingivalis*. Intestinal contents were obtained at 1, 3, and 16 hrs after administration of *P. gingivalis*. DNA was extracted from whole blood and intestinal contents using a QIAampDNA Blood Mini Kit (Qiagen, Hilden, Germany) and QIAamp DNA Stool Mini Kit (Qiagen), respectively. Quantitative real-time PCR was performed on a LightCycler® 96 System (Roche) using Fast Start Essential DNA Green Master (Roche). Universal 16S rRNA was amplified using forward primer 5′-ACTCCTACGGGAGGCAGCAGT-3′ and reverse primer 5′-ATTACCGCGGCTGCTGGC-3′.

*P. gingivalis* 16S rRNA was amplified using forward primer 5′-AGGCAGCTTGCCATACTGCG-3′and reverse primer 5′- ACTGTTAGCAACTACCGATGT-3′.

### Gut microbiota analysis by pyrosequencing

The ileum was removed and the ileal contents were collected using sterile water (Bio-Rad Laboratories, Hercules, CA). The obtained ileal contents were homogenised and DNA was extracted using a PowerSoil® DNA isolation kit (MO BIO, Carlsbad, CA). PCR was performed using a primer set (784F:5′-AGGATTAGATACCCTGGTA-3′ and 1061R: 5′-CRRCACGAGCTGACGAC-3′) targeting the V5-V6 region of the 16S rRNA genes^60^. To amplify the targeted region, 1 μl of extracted DNA served as the template in 50-μl reactions using KAPA HiFi HS Ready Mix (KAPA Biosystems, Woburn, MA). The PCR protocols were 95°C for 3 min, 25 cycles of 98°C for 20 s, 60°C for 15 s, and 72°C for 15 s and 72°C for 1 min. Two 100 μl of 3-cycle reconditioning PCR reactions were performed per sample to eliminate heteroduplexes, with 10-μl aliquots of the initial PCR product mixture as the template and other PCR conditions unchanged. Products of the two reconditioning PCR reactions per sample were combined and purified using DNA clean and Concentrator-5 (ZYMO RESEARCH, Irvine, CA). Pyrosequencing of the 16S rRNA amplicons was carried out on the 454 GS Junior platform (Roche, Basel, Switzerland). Denoising, taxonomic assignments, and estimating relative abundance of sequencing data were performed by the analysis pipeline of the QIIME software package^61^. An operational taxonomic unit (OTU) was defined at 97% similarity. OTU indicating relative abundance of under 0.1% was filtered to remove noise.

### Glucose and insulin tolerance tests

GTTs were performed in mice orally administered *P. gingivalis* and sham-administered mice. Mice were injected i.p. with a single dose of 1 g of glucose per kg of body weight. For the insulin tolerance test, insulin (0.5 unit/kg) was administered by an i.p. injection. Blood samples were collected through the tail vein before glucose or insulin injection and at 15, 30, 60, 90, and 120 min. Blood glucose concentrations were immediately determined by the Glucose Pilot assay (Aventir Biotech, LLC CA). The serum insulin concentration was determined for by ELISA (Morinaga, Tokyo, Japan).

### Periodontal tissue, liver and adipose tissue histology

The fixed mandibles were dissected, decalcified, embedded, and sectioned as described previously^62^. Serial sections 5 μm thick were obtained in the sagittal direction along the long axis of the teeth and stained with hematoxylin and eosin. Mouse livers were embedded in Tissue-Tek OCT (Finetechnical Sakura, Tokyo, Japan) and frozen in liquid nitrogen. Frozen 7 μm sections were obtained in a cryostat (LEICA, Wetzlar, Germany), dried at room temperature for 60 min, fixed in 10% formaldehyde for 10 min, stained with oil red O for 20 min, and counter-stained with hematoxylin.

Adipose tissue samples from sham-administered and *P. gingivalis*-administered mice were fixed in 10% formalin for immunohistochemistry. Briefly, samples were embedded in paraffin, sectioned, and stained with rat anti-mouse F4/80 antibody (ABd Serotec, Raleigh, NC; 1:50 dilution).

### Cytokine assay

Proteins were extracted using T-PER Mammalian Protein Extraction Reagent (Pierce Biotechnology, Rockford, IL). C1q/TNF-related protein 9 (CTRP9) and TNF-α were measured using the Mouse CTRP9 ELISA kit (Aviscera Bioscience, Santa Clara, CA) and the Mouse TNFα ELISA kit (Thermo Scientific, Seattle, WA), respectively. The total protein concentrations were measured by the Pierce BCA Protein Assay kit (Thermo Scientific). Individual protein concentrations were calculated as the abundance of specific protein constituents divided by the total protein concentrations. Serum IL-6 level was determined by using a commercial ELISA kit (Thermo Scientific).

### Endotoxin assay

Endotoxin levels were determined in sera collected at 16 hrs after final administration of *P. gingivalis* from the tail veins of mice using a limulus amoebocyte lysate test (QCL-1000TM, BioWhittaker, Walkersville, MD) according to the manufacturer's instruction. Serum samples were diluted at 1 to 4 for the assay. Optical densities were measured using an ELISA plate reader (Model 680, Bio-Rad Laboratories) at 405 nm.

### Liver triglyceride content

Liver triglycerides were determined with a Triglyceride Quantification Colorimetric kit (Bio Vision, Milpitas, CA). Triglyceride values were expressed as the concentration of triglycerides divided by the total protein concentration. The total protein concentrations were measured by the Pierce BCA Protein Assay kit (Thermo Scientific).

### Analysis of gene expression in adipose tissue, liver, and intestine

Total RNA from adipose tissue, liver, small intestine and large intestine samples was extracted using an RNeasy Mini kit and treated with DNase I (Qiagen, Germantown, MD) according to the manufacturer's instructions. Aliquots of RNA were then reverse-transcribed to cDNA using random primers (Takara Bio Inc., Shiga, Japan) and M-MLV reverse transcriptase (Life Technologies Corporation, Carlsbad, CA). Primers and probes specific for real-time PCR were purchased from Life Technologies Corporation. Reactions were carried out in a 25-μl mixture in a LightCycler® 96 System(Roche) using TaqMan Gene Expression Assays (Life Technologies Corporation) containing a 900 nM primer and a 250 nM probe. The reactions consisted of a 10-minute incubation at 95°C, followed by 40 cycles of a two-step amplification procedure consisting of annealing/extension at 60°C for 1 minute and denaturation for 15 seconds at 95°C. LightCycler® 96 software (Roche) was used to analyze the standards and carry out the quantifications. The relative quantity of each mRNA was normalized to the relative quantity of glyceraldehyde-3-phosphate dehydrogenase (GAPDH) mRNA.

### Statistical analysis

Nonparametric data were evaluated using the Mann-Whitney U-test for two-group comparisons, whereas comparisons of three or more groups were analysed by one-way ANOVA with ad hoc Bonferroni post tests using Graphpad Prism® (GraphPad Software, Inc., La Jolla, CA). A probability value of p < 0.05 was considered statistically significant. For assessing significant differences between the *P. gingivalis*-administered and sham-administered samples, we used the Mann–Whitney U-test from the R package (http://cran.at.r-project.org/).

## Author Contributions

K.A. researched data and wrote the manuscript. H.Y., H.M., T.M., M.N., K.G. and D.M. researched data. S.N. researched data and contributed to discussion. M.R. and T.I. contributed to discussion and reviewed/edited manuscript. K.Y. wrote the manuscript. All authors reviewed the manuscript.

## Supplementary Material

Supplementary Information Supplementary information
