# Supplementary material for: Oral pathobiont induces systemic inflammation and metabolic changes associated with alteration of gut microbiota
Source: Sci Rep. 2014 May 6;4:4828. doi: 10.1038/srep04828 (PMC4010932; doi:10.1038/srep04828)
Supplement: Supplementary Information [file srep04828-s1.pdf]

Supplementary information

Oral pathobiont induces systemic inflammation and metabolic changes associated with alteration of gut microbiota

Kei Arimatsu[1,2], Hitomi Yamada[1,2], Haruna Miyazawa[1,2], Takayoshi Minagawa[1,2], Mayuka Nakajima[1,2], Mark I. Ryder[3], Kazuyoshi Gotoh[4], Daisuke Motooka[4], Shota Nakamura[4], Tetsuya Iida[4] and Kazuhisa Yamazaki[1*]

[1]Laboratory of Periodontology and Immunology, Division of Oral Science for Health Promotion, Niigata University Graduate School of Medical and Dental Sciences, Niigata, Japan

[2]Division of Periodontology, Department of Oral Biological Science, Niigata University Graduate School of Medical and Dental Sciences, Niigata, Japan

[3] Division of Periodontology, Department of Orofacial Sciences, School of Dentistry, University of California, San Francisco, USA

[4]Department of Infection Metagenomics, Research Institute for Microbial Diseases, Osaka University, Osaka, Japan

[*]Correspondence: Prof. Kazuhisa Yamazaki, Laboratory of Periodontology and Immunology, Division of Oral Science for Health Promotion, Niigata University Graduate School of Medical and Dental Sciences, 5274 Gakkocho 2-ban-cho, Chuo-ku, Niigata 951-8514, Japan

Tel: +81-25-227-0744, Fax: +81-25-227-0744, E-mail: kaz@dent.niigata-u.ac.jp

Supplementary Figure S1

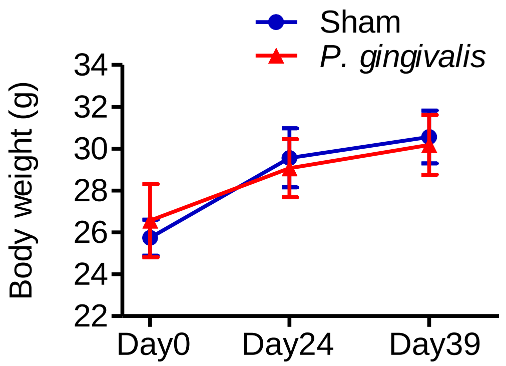

Changes in body weight during experimental period (N = 8 in each group).

Supplementary Figure S2

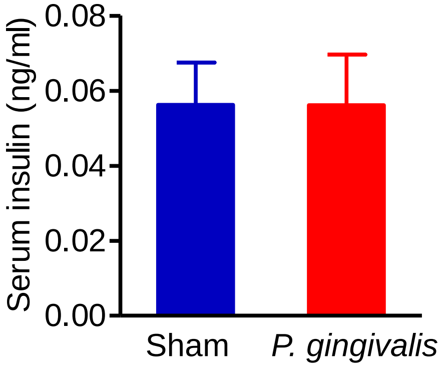

Insulin levels of *P. gingivalis*-administered and sham-administered mice. Fasting serum insulin level were determined by ELISA (N = 10 in each group).

Supplementary Figure S3

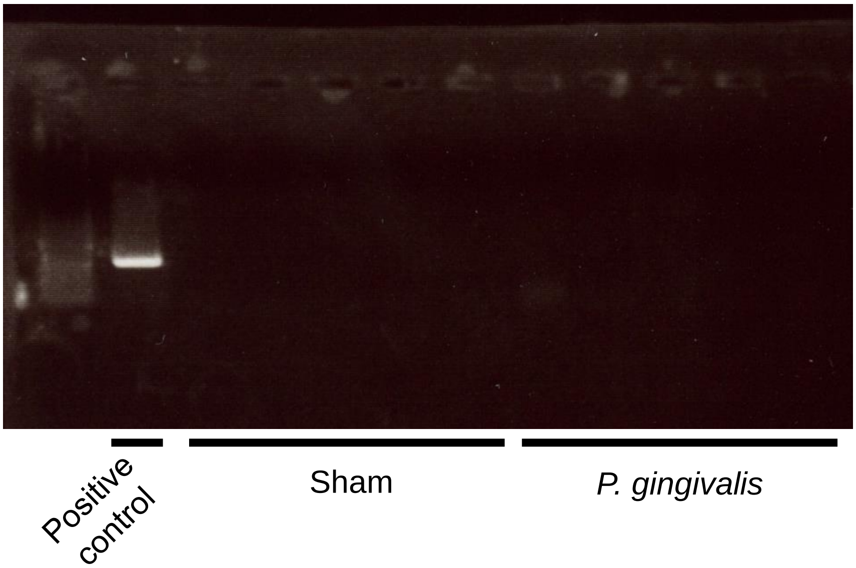

Detection of *P. gingivalis* in the ileal samples of *P. gingivalis*-administered and sham-administered mice. Representative 1.2% agarose gels showing the results of PCR amplification of DNA extracted from ileal samples for detection of *P. gingivalis* 16S rRNA.

Supplementary Figure S4

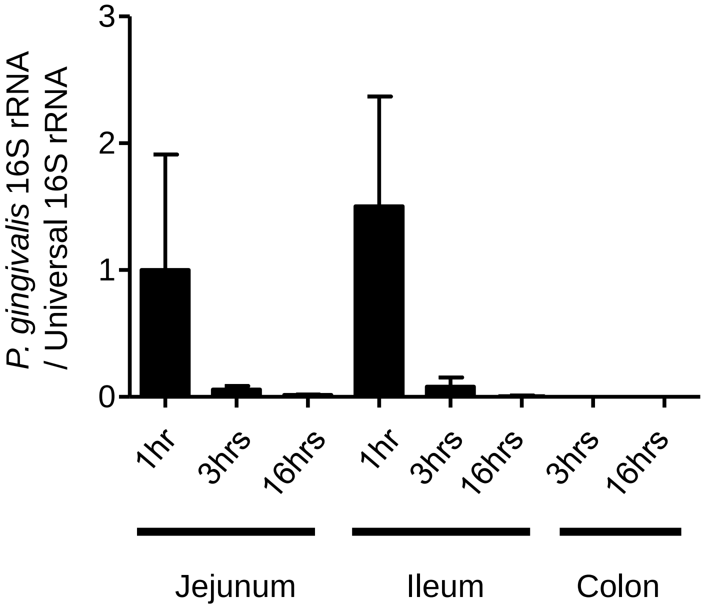

Translocation of administered *P. gingivalis* in the intestinal tract. After a single administration of *P. gingivalis*, intestinal contents were recovered from the jejunum, ileum, and colon at 1, 3, and 16 hrs. Relative abundance of *P. gingivalis*-specific 16S rRNA gene to universal 16S rRNA genes are shown.

Supplementary Figure S5

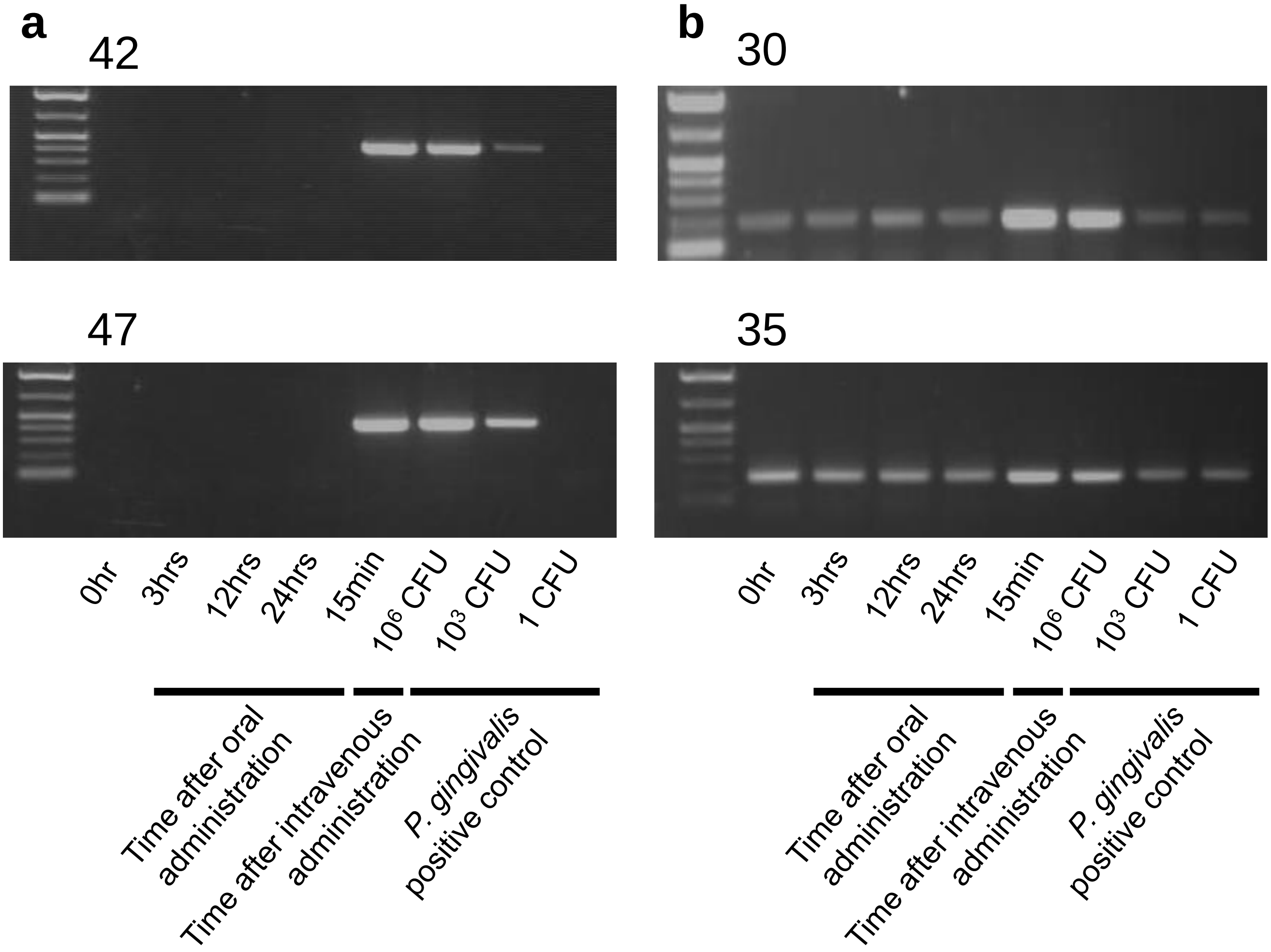

Detection of *P. gingivalis*-specific 16S rRNA gene (a) or universal 16S rRNA genes (b) in the blood samples of *P. gingivalis*-administered and sham-administered mice. After a single administration of *P. gingivalis* orally or intravenously (infraorbital vein), blood draws were taken from left ventricle of the heart at 0, 3, 12, and 24 hrs or 15 min, respectively. Representative results of one of the three independent experiments are shown. The number of PCR cycles is shown above lanes.
